# The impact of work-place social capital in hospitals on patient-reported quality of care: a cohort study of 5205 employees and 23,872 patients in Denmark

**DOI:** 10.1186/s12913-021-06498-x

**Published:** 2021-05-31

**Authors:** Alice Clark, Thim Prætorius, Eszter Török, Ulla Arthur Hvidtfeldt, Peter Hasle, Naja Hulvej Rod

**Affiliations:** 1 Department of Public Health, University of Copenhagen, Copenhagen, Denmark; 2 Steno Diabetes Center Aarhus, Aarhus University Hospital, Aarhus, Denmark; 3 Danish Cancer Society Research Center, Copenhagen, Denmark; 4 Department of Technology and Innovation, University of Southern Denmark, Odense, Denmark

## Abstract

**Background:**

Decision-makers increasingly consider patient-reported outcomes as important measures of care quality. Studies on the importance of work-place social capital–a collective work-place resource–for the experience of care quality are lacking. We determined the association between the level of work-place social capital and patient-reported quality of care in 148 hospital sections in the Capital Region of Denmark.

**Methods:**

This cross-sectional study combined section-level social capital from 5205 health care professionals and 23,872 patient responses about care quality. Work-place social capital encompassed three dimensions: trust, justice and collaboration. Patient-reported quality of care was measured as: overall satisfaction, patient involvement, and medical errors. Linear regression analysis and generalized linear models assessed the mean differences in patient reported experience outcomes and the risk of belonging to the lowest tertile of care quality.

**Results:**

A higher level of work-place social capital (corresponding to the interquartile range) was associated with higher patient-reported satisfaction and inpatient and acute care patient involvement. The risk of a section belonging to the lowest tertile of patient involvement was lower in sections with higher social capital providing inpatient (RR = 0.39, 0.19–0.81 per IQR increase) and acute care (RR = 0.53, 0.31–0.89). Patient-reported errors were fewer in acute care sections with higher social capital (RR = 0.65, 0.43 to 0.99). The risk of being in the lowest tertile of patient-reported satisfaction was supported for acute care sections (RR = 0.47, 0.28–0.79).

**Conclusions:**

Although we found small absolute differences in the association between patient-reported experience measures and social capital, even a small upward shift in the distribution of social capital in the hospital sector would, at the population level, have a large positive impact on patients’ care experience.

**Supplementary Information:**

The online version contains supplementary material available at 10.1186/s12913-021-06498-x.

## Introduction

Hospitals must increase efficiency and quality of care to meet growing patient demands and expectations [1]. To these ends, hospitals restructure how they deliver health care [2] by introducing management systems such as lean [3] or value-based health care [4], merging departments or hospitals, [5] or by implementing hospital-wide IT enterprise systems [6]. However, restructuring how hospitals operate at the frontline [7, 8] may reduce health care professionals’ psychosocial work environment and well-being [9] known to influence organisational performance [10]. To maintain a good psychosocial work environment and wellbeing under circumstances of change and uncertainty, research within and outside the health care domain finds that having a high level of social capital is important [11, 12]. Social capital is an inter-personal phenomenon [13] consisting of trust, justice, and collaboration components that inheres in the structure of relations between actors and among actors [14]. It may help to foster work-place participation, reciprocity, and interpersonal trust [11] that in turn allow employees to collaborate about solving complex organisational tasks [15] for mutual benefit and with higher performance [16].

Delivering efficient and safe health care is a complex task requiring interdisciplinary collaboration, to which end health care researchers have studied social capital [17] and the related measure of relational coordination [18]. This literature finds that higher levels of social capital improve care coordination [19], patient care [20, 21] and safety [22], and reduces the length of stay [23]. For health care professionals, social capital improves job satisfaction [24–26], work engagement [24, 27], knowledge sharing [22] and innovation adoption [28], and reduces burnout [21, 29], long-term sickness absence [30], and work-home conflict [31]. At the hospital level, social capital is associated with better quality management systems [32] and clinical risk management [33]. Nevertheless, research studying the effect of social capital on patient-reported experience measures remain scarce [34] even though they represent vital measures of health care performance [35, 36] because the patient is uniquely positioned to assess the experienced care quality [37]. Despite social capital being an inter-personal phenomenon, previous studies also typically investigate individual-level rather than group-level social capital, thereby limiting our understanding of the extent to which the aggregate, collective level drives care and patient-reported outcomes.

Given these knowledge gaps, this article aims to determine the relationship between work-place social capital and patient-reported quality of care. For the purpose of this article, we operationalise patient-reported care quality as patient’s overall satisfaction with their acute, in- or outpatient care, level of perceived involvement in decision processes, and occurrence of patient-reported medical errors, thereby studying three central parameters of health care performance. We hypothesise that a higher level of social capital in a hospital section is associated with higher patient satisfaction, greater involvement of patients, and fewer medical errors. To test this hypothesis, we use survey data from 5205 employees and 23,872 patients from 148 hospital sections spanning 16 health care institutions serving 1.8 million citizens in the Capital Region of Denmark.

## Methods

### Study population

#### The Well-being in HospitAL Employees cohort

To capture the psychosocial work environment of health care workers, we identified eligible hospital sections by using data from the Well-being in HospitAL Employees (WHALE) cohort [38]. The WHALE cohort initiated in 2011 and followed up with new waves in 2014 and 2017 contains survey information from hospital employees within the Capital Region of Denmark about their physical and psychosocial work environment and detailed information on the organisational structure (including organisation, department and section). In 2014, 37,720 health care employees working at 1089 hospital sections within the Capital Region of Denmark were invited to the survey. Of that number, 84% participated by filling in a self-reported questionnaire. These data allowed for, first, conducting the analysis of social capital at an aggregated level and, second, linking the aggregated measure with patient-reported quality at the same organisational level.

#### The national survey of patient experiences

The Unit for Evaluation and User-involvement (UEU) in Denmark conducts yearly surveys of patients’ experiences of the care received across inpatient, outpatient, and acute sections in all Danish hospitals (see [39] for a detailed elaboration). In 2014, our sample year, the UEU invited a random selection of patients seen in the different hospital sections between August 1 and October 31. Up to 400 patients were included from each hospital section: in case fewer were seen within the inclusion period, the UEU invited all attending patients. Data were collected from all hospital sections except physiotherapy, occupational therapy, anaesthesiology, radiology, diagnostic imaging, biochemistry, physiology, and nuclear medicine because they mostly provided services to other sections that had the primary patient responsibility.

#### Data selection

**Fig. 1:**
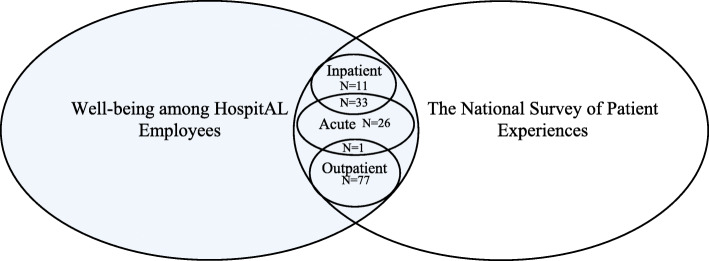
The identifiability of hospital sections in the Well-being among HospitAL Employees cohort and the National Survey of Patient Experiences in the Capital Region of Denmark

Data was derived largely from administrative databases reducing issues of missing information. Since only aggregated measures were used, single missing values for individual employees or patients did not exclude these cases from the data. However, information at the aggregated level was considered for analysis only if > 50% of participants at a given section provided valid information. Due to differences in the hierarchal administrative organisation of sections in WHALE and the national survey of patient experiences (NSPE), not all sections from the latter data set were identifiable in the former. We identified 69 out of 100 sections providing acute care, 51 out of 79 sections of inpatient care, and 89 out of 193 sections providing outpatient care in both data sets. To preserve anonymity for smaller sections or sections with only few patient reports, we excluded sections with < 3 employees (3 sections) or < 10 patient reports of quality of care (19 sections). In addition, one section was excluded due to insufficient data on social capital or covariates. This resulted in a total study sample of 148 unique hospital sections, 34 of which had an overlap in the care they provided: 44 provided inpatient care, 60 acute care, 78 sections provided outpatient care, 33 sections provided both acute and inpatient care, and one section provided both acute and outpatient care. For the purpose of our analysis, we used the combined total of 182 hospital sections, thus including the 34 sections proving care in two contexts in both of those study samples. The identifiability and overlap of sections in the WHALE and NSPE cohorts are illustrated in Fig. 1. Across nine hospitals in the Capital Region of Denmark, we included a total of 5205 employee responses about social capital measures and 23,872 patient responses about their perceived quality of care. Patients reporting from hospital sections that could not be identified in WHALE differed in some regards (Additional file 1: Appendix 1). Patients in the unidentifiable hospital sections were more likely to be women and < 60 years of age and they reported receiving a slightly higher average quality of care, more outpatient care and less acute care, and the acute and outpatient care they received were more likely to exceed 24 h.

### Measures

#### Social capital

Social capital was measured by eight items [40] covering the three dimensions of trust, justice, and collaboration between employees at the same hierarchical level and with their manager(s) at higher hierarchical levels [38]. Examples of items of the three dimensions are, respectively: ‘Do you trust the information that comes from the management?’, ‘Is the work distributed fairly?’, and ‘Do you and your colleagues take responsibility for creating a nice atmosphere and tone of communication?’ The full list of items is available in Additional file 1: Appendix 2. The response categories were on 5- and 7-point Likert scales ranging from ‘not at all’ to ‘to a very large extent’, which we converted into a scale ranging from 0 to 100 to be able to aggregate answers. We measured individual-level social capital as the mean of the converted item responses with higher scores representing higher social capital. Individual-level social capital was recorded as missing if the employee responded to fewer than four of the eight items. To measure social capital at the section level, we aggregated the mean of individual-level social capital within each hospital section. Section-level social capital was recorded as missing if data were available for < 50% of employees. Similar operationalisations of aggregated social capital have previously been applied within the WHALE cohort, and we have estimated the Cronbach’s alpha coefficient of the social capital scale to be 0.85 for the 2014 survey.

#### Patient-reported quality of care

We used distinct surveys covering the same three dimensions of quality of care that were collected from the three groups (inpatient, outpatient, and acute care) as part of the NSPE [33]. *Overall satisfaction* was measured by three items concerning patients’ satisfaction with treatment, care, and the experience as a whole. *Patient involvement* was measured by five items. Three items concerned the extent to which the health care staff asked the patient about their experiences with the disease, talked to the patient about disease self-management, and considered the patient’s needs when planning the discharge (not including outpatient care). Two items concerned the extent to which the patient and next of kin had the opportunity to take part in shared decision-making. Response categories for overall satisfaction and patient involvement were on a 5-point Likert scale from ‘to a very large extent’ to ‘not at all’. Overall satisfaction and patient involvement were operationalised as the mean level of patients’ responses (range 1–5) with higher values reflecting a more positive evaluation. *Medical errors* were measured by patients’ reporting of the occurrence of errors during their stay (yes/no). The included scales of patient-reported quality of care have previously been found reliable using item response theory models within the Danish patient population [41].

The average quality of care was aggregated within hospital sections based on the mean values of patients’ replies to the items measuring satisfaction and patient involvement. A patient observation was included in the analysis if they had provided a minimum of two and three responses (two for outpatient cares) to the included satisfaction and involvement dimensions. An aggregated measure of section-level quality was included if > 50% of patients provided valid responses regarding a given dimension. Medical errors were measured as the average reported occurrence of errors among patients attending each hospital section.

### Covariates

Both patient and hospital section characteristics were included as covariates. Information on the patient composition within each hospital section included the distribution of age, gender, and length-of-stay that were measured as aggregated measures of patients’ responses. Using data obtained from administrative databases at the Capital Region of Denmark, we included relevant hospital section characteristics: affiliated hospital, size, complexity, proportion of employees on long-term sick-leave, and use of part-time workers (see below for an elaboration of how these were operationalised).

### Statistics

Descriptive analyses were carried out to assess the mean level of patient satisfaction, patient involvement and occurrence of medical errors at the section-level according to hospital section characteristics of both patient and employee compositions.

To determine the relation between section-level social capital and patient-reported quality of care, we used an ordinary least square linear regression analysis to assess the mean differences in level of patient satisfaction, patient involvement, and occurrence of medical errors. To determine the relation between section-level social capital and the risk of being in the lowest tertile of patient-reported quality of care, we used generalized linear models with a Poisson distribution and log link rather than a logistic model because of the high prevalence of the outcome. This also enabled the direct estimation of risk ratios. Confidence intervals were estimated by using robust error variances. The lowest tertile was selected as the low patient-reported quality of care group due to the distribution of the outcomes, where a lower threshold would have resulted in unstable analyses due to few observations.

**Fig. 2:**
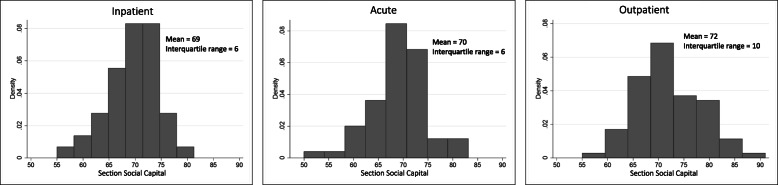
Distribution of hospital section social capital according to care group

The full distribution of section-level social capital in hospital sections providing acute, in- and outpatient care, respectively, is displayed in Fig. 2. All associations between section-level social capital and patient-reported quality of care were assessed using the interquartile range (IQR) of social capital in each of the three types of care sections. We used the IQR as a metric of change because it allowed us to capture the impact of shifting a section from the lowest (25% cut-off point) to the highest quartile (75% cut-off point) of social capital. For the three types of sections the IQR was six, six and ten for, respectively, inpatient, acute and outpatient care, meaning that the effects estimated correspond to an improvement in section social capital of this magnitude.

The IQR was similarly used in the adjusted analyses where each IQR-unit increase in social capital was analysed to determine the association with the three outcome measures. The analyses were adjusted for potential confounding from section-level information on both patient and employee compositions. Potential patient composition confounders comprised: gender (proportion of female patients), age (proportion ≥ 60 years of age), and length-of-stay (proportion receiving care for < 24 h, not including outpatient care). Potential employee composition confounders comprised: hospital, hospital section size (number of active employees, i.e., not absent due to parental leave or education), complexity (number of employee-defined smaller work-units, i.e., employees referring to the same manager (described elsewhere: 9, 30), proportion of part-time workers (< 37 h/week), and proportion of employees on long-term sick-leave (exceeding 29 days within one year before assessment). We analysed the three patient groups (inpatient, outpatient, and acute) separately because of the different ways in which care is delivered across these functions [42].

Sensitivity analyses were performed to determine if effects could be ascribed to any of the three components of social capital (trust, justice or collaboration) by analysing each aspect separately.

## Results

### Hospital section and patient characteristics

Table 1 shows mean levels of patient-reported satisfaction, involvement, and the occurrence of medical errors according to patient and hospital section characteristics.

**Table 1:** Patient-reported quality of care according to patient and section characteristics of the included hospital sections

	Number (%)	Satisfaction^a^ Mean (SD)	Patient involvement^a^ Mean (SD)	Medical errors^a^ Mean proportion (SD)
**Patient characteristics**				
**Type of care**				
Inpatient	4388 (18)	4.29 (0.78)	3.71 (1.07)	0.10 (0.29)
Acute	5878 (25)	4.00 (0.98)	3.22 (1.14)	0.13 (0.33)
Outpatient	13,606 (57)	4.27 (0.82)	3.61 (1.07)	0.05 (0.22)
**Gender**				
Women	12,686 (53)	4.16 (0.89)	3.51 (1.15)	0.08 (0.27)
Men	11,186 (47)	4.26 (0.82)	3.65 (1.05)	0.07 (0.26)
**Age**				
< 60	9628 (40)	4.16 (0.90)	3.63 (1.07)	0.09 (0.29)
≥ 60	14,244 (60)	4.24 (0.83)	3.54 (1.13)	0.06 (0.25)
**Length-of-stay**^c^				
< 24 h	4543 (44)	4.12 (0.94)	3.44 (1.12)	0.10 (0.30)
≥ 24 h	5723 (56)	4.13 (0.89)	3.35 (1.12)	0.12 (0.33)
**Section characteristics**				
**Type of care**				
Acute	26	3.96 (0.20)	3.96 (0.20)	0.13 (0.061)
Inpatient	11	4.29 (0.24)	4.29 (0.24)	0.067 (0.057)
Outpatient	77	4.27 (0.16)	4.27 (0.16)	0.048 (0.029)
Acute and inpatient	33	4.23 (0.18)	4.23 (0.18)	0.10 (0.040)
Acute and outpatient	1	4.17	3.71	0.022
**Social capital**^b^				
Low < 25 percentile (< 67)	46	4.19 (0.24)	3.56 (0.071)	0.093 (0.071)
Medium 25–75 percentile (67–74)	95	4.16 (0.25)	3.49 (0.39)	0.092 (0.056)
High > 75 percentile (> 74)	41	4.25 (0.13)	3.72 (0.22)	0.064 (0.041)
**Part-time workers** (according to the median)^b^				
< 56%	90	4.18 (0.24)	3.57 (0.35)	0.087 (0.056)
≥ 56%	92	4.19 (0.22)	3.56 (0.35)	0.085 (0.061)
**Sickness absence 1 yr. prior** (according to the median)^b^				
< 5%	89	4.23 (0.21)	3.63 (0.33)	0.080 (0.066)
≥ 5%	93	4.15 (0.24)	3.50 (0.37)	0.092 (0.050)
**Section size** (according to tertiles)^b^				
< 18 employees	51	4.23 (0.18)	3.72 (0.29)	0.060 (0.042)
18–34 employees	82	4.20 (0.24)	3.56 (0.35)	0.091 (0.064)
> 34 employees	49	4.12 (0.25)	3.40 (0.36)	0.11 (0.053)
**Section complexity** (number of work-units) (according to tertiles)^b^				
< 3	53	4.22 (0.21)	3.67 (0.33)	0.075 (0.051)
3–4	63	4.22 (0.19)	3.60 (0.32)	0.081 (0.060)
> 4	66	4.13 (0.26)	3.44 (0.38)	0.099 (0.061)

^a^ Range of the care quality scales:1–5. ^b^ according to the distribution among the 148 unique sections. ^c^ not relevant for outpatient care

The mean level of patient-reported quality of care was generally lower for patients receiving acute care than those receiving inpatient or outpatient care. The level of reported patient involvement was lower for patients staying for more than 24 h or who were 60 years of age or older. Older patients, however, were generally more satisfied with their care and less likely to report medical errors. Women and men reported similar mean levels of satisfaction, involvement and occurrence of errors.

Regarding hospital section characteristic, those providing acute care had lower patient-reported satisfaction, involvement and higher medical errors than those providing inpatient or outpatient care. Patient-reported quality of care was slightly higher for hospital sections within the highest quartile of social capital, although the pattern was not consistent across low, medium, and high levels of social capital. Slightly lower levels of patient-reported quality of care were also found for hospital sections with higher rates of long-term sickness absence among employees, a larger number of employees, and higher section complexity (i.e. number of work-units). The use of part-time workers resulted in similar levels of patient-reported quality of care.

### Patient satisfaction

**Fig. 3:**
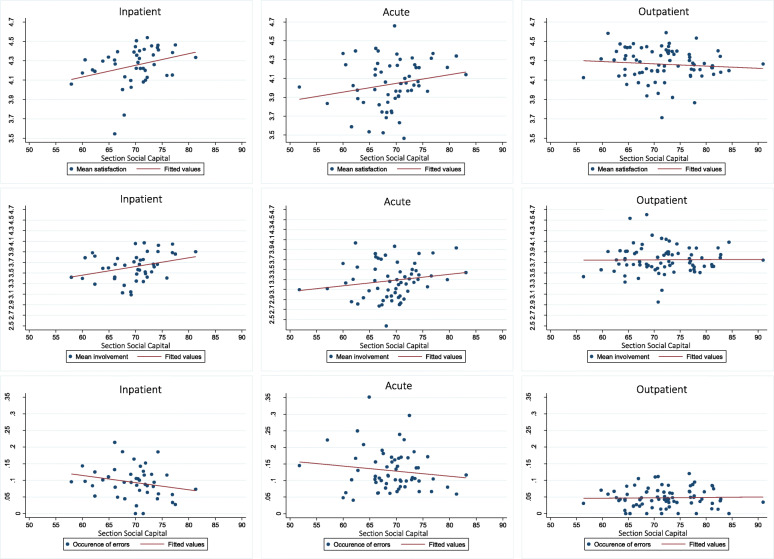
The correlation between section social capital and patient-reported quality of care

Generally, the majority of patients reported a high degree of satisfaction with their care. A larger variation in the mean level of satisfaction was seen among hospital sections providing acute care compared to inpatient and outpatient care (Fig. 3). The positive correlation between section-level social capital and mean level of satisfaction among inpatient and acute care seen in Fig. 3 was also found in the adjusted analyses (inpatient: 0.09 (0.00, 0.19), acute: 0.08 (0.01, 0.15) presented in Table 2. However, the association between section-level social capital and risk of a hospital section being in the lowest tertile of patient-reported satisfaction was supported only for acute care, with approximately half the risk associated with an IQR increase in social capital (RR = 0.47; 95% CI: 0.28 to 0.79) (Table 3).

**Table 2:** Section social capital and mean differences in patient-reported quality of care in 148 hospital sections

	Inpatient care	Acute care	Outpatient care
Number of hospital sections	44	60	78
Mean (IQR) hospital section social capital	69 (6)	70 (6)	72 (10)
**Mean patient satisfaction**			
Mean level of patient satisfaction (SD)	4.25 (0.19)	4.04 (0.25)	4.26 (0.16)
Multiple adjusted^a^ mean differences (95% CI) according to IQR of hospital section social capital	**0.09 (0.00, 0.19)**	**0.08 (0.01, 0.15)**	−0.01 (−0.08, 0.05)
**Mean patient involvement**			
Mean level of patient involvement (SD)	3.62 (0.26)	3.28 (0.35)	3.75 (0.26)
Multiple adjusted^a^ mean differences (95% CI) according to IQR of hospital section social capital	**0.15 (0.03, 0.27)**	0.08 (−0.01, 0.18)	0.02 (−0.07, 0.12)
**Mean occurrence of medical errors**			
Mean occurrence of medical errors (SD)	0.09 (0.05)	0.13 (0.06)	0.05 (0.03)
Multiple adjusted^a^ mean differences (95% CI) according to IQR of hospital section social capital	−0.01 (−0.04, 0.02)	−0.01 (− 0.03, 0.01)	0.01 (− 0.01, 0.02)

^a^Adjusted for section characteristics: Hospital, number of employees, complexity, mean age of employees, proportion of females, proportion of part-time employees, proportion with prior LTSA, patient characteristics: proportion females, proportion 60 years or older, proportion with length-of-stay exceeding 24 h

Bold numbers indicate statistical significance at the 0.05 level

**Table 3:** Section social capital and risk ratios (95% CI) of being in the lowest tertile of patient-reported quality of care in 148 hospital sections

	Inpatient care	Acute care	Outpatient care
Number of hospital sections	44	60	78
Mean (IQR) hospital section social capital	69 (6)	70 (6)	72 (10)
**Patient satisfaction**			
Lowest 33-percentile cut-point of patient satisfaction	4.19	3.96	4.20
Multiple adjusted^a^ RR (95% CI) according to IQR of hospital section social capital	0.63 (0.31, 1.28)	**0.47 (0.28, 0.79)**	0.80 (0.46, 1.38)
**Patient involvement**			
Lowest 33-percentile cut-point of patient involvement	3.49	3.10	3.63
Multiple adjusted^a^ RR (95% CI) according to IQR of hospital section social capital	**0.39 (0.19, 0.81)**	**0.53 (0.31, 0.89)**	0.93 (0.52, 1.67)
**Medical errors**			
Highest 33-percentile cut-point of the occurrence of medical errors	0.11	0.14	0.06
Multiple adjusted^a^ RR (95% CI), according to IQR of hospital section social capital	0.87 (0.49, 1.54)	**0.65 (0.43, 0.99)**	1.26 (0.73, 2.18)

^a^Adjusted for section characteristics: Hospital, number of employees, complexity, mean age of employees, proportion of females, proportion of part-time employees, proportion with prior LTSA, patient characteristics: proportion females, proportion 60 years or older, proportion with length-of-stay exceeding 24 h

Bold numbers indicate statistical significance at the 0.05 level

RR < 1 means that the risk of the outcome (e.g., low patient involvement) decreases with exposure (i.e., higher social capital)

### Patient involvement

The mean level of reported patient involvement was between 3 (to some extent) and 4 (to a high extent), with the level being slightly lower for acute care. As Fig. 3 shows, there was a positive correlation between section-level social capital and the mean level of patient involvement in hospital sections providing acute or inpatient care. Yet, in the adjusted analyses the association was apparent only for inpatient care, where each 6-unit increase in social capital was associated with a 0.15 (0.03, 0.27) higher mean level of patient involvement (Table 2). The risk of a hospital section belonging to the lowest tertile of mean patient involvement was lower in sections with high social capital among both inpatient (RR = 0.39, 95% CI: 0.19 to 0.81) and acute care (RR = 0.53, 95% CI: 0.31 to 0.89) (Table 3).

### Medical errors

The mean occurrence of medical errors reported by patients varied between the three types of care, with a higher occurrence and variation in acute than inpatient and outpatient care, with reported proportions of 13, 9, and 5%, respectively (Fig. 3). The weak negative correlation between section-level social capital and the mean occurrence of medical errors seen in Fig. 3 for acute and inpatient care was not supported in the adjusted analyses (Table 2). The risk of being in the tertile of hospital sections with the highest occurrence of patient-reported errors was lower in sections with higher levels of social capital caring for acute patients (RR = 0.65, 95% CI: 0.43 to 0.99) while there was no association for inpatient and outpatient care (Table 3).

### Sensitivity analyses

The adjusted analysis of the correlations between the three components of social capital (trust, justice, and collaboration) with each of the three measures of quality of care (Additional file 1: Appendix 3a-c) show patterns similar to the main analysis based on the combined measure of section-level social capital, but with a larger variance. In terms of absolute differences, the adjusted effect estimates point in the same direction as the main analysis, but the greater variance results in wider confidence intervals (Additional file 1: Appendix 4).

The association in the main analysis between section-level social capital and the risk of being in the lowest tertile of patient-reported quality was partly supported when analysing the three components separately (Additional file 1: Appendix 5). A statistically significant association was found between section-level trust and patient satisfaction and between section-level justice and patient involvement for inpatient care. For acute care a statistically significant association was found between section-level trust and patient satisfaction, between section-level justice and patient satisfaction and patient involvement, and between section-level collaboration and patient involvement. No associations were found for outpatient care regarding the separate dimensions.

## Discussion

The study investigated the association between section-level social capital and the level of patient-reported quality of care among 148 Danish hospital sections spanning acute, inpatient, and outpatient care delivery. Our research extends the existing literature on work-place social capital by analysing the effect of social capital on patient-reported outcomes, an outcome measure increasingly seen as a vital indicator of health care performance. We found that a higher level of section-level social capital in hospitals was associated with higher patient-reported satisfaction and involvement for inpatient and acute care but not for outpatient care. Although the absolute differences found in our study are small, elevated to the entire population of health care professionals and patients, even a small upward shift in the distribution of social capital in the hospital sector would improve the care delivered and experienced by patients.

Studying the association with patient-reported outcomes at the section-level renders it possible to study the interpersonal phenomenon of social capital at the relevant group level where health care professionals together deliver health care and jointly contribute to the patient experience. By measuring social capital at the aggregated group level, we add to the current literature on social capital and performance outcomes that do not adequately consider the former as an interpersonal phenomenon. Moreover, the current evidence base relies predominantly on reporting by the health care professionals and not the receiver of the care. Compared to previous studies, our study more fully adjusts for two sets of covariates known to influence how hospitals operate and how health care professionals deliver care. Analyses were adjusted for, first, the level of organisational complexity (operationalised as the section size and number of work units in a section) and, second, the type of work environment (operationalised as the proportion of employees with prior long-term sick-leave and use of part-time workers). Similar to our study, earlier studies also find that workplace social capital is an organisational resource that leads to better care outcomes such as length-of-stay [18, 23], care quality [20, 21], and patient safety [22] and a better work environment as measured by job satisfaction [24–26], work engagement [24, 27], knowledge sharing [22], burnout [21, 29], and long-term sickness absence [30].

The present analysis benefits from relying on a large data set of systematically collected register data. Data on workplace social capital came from a large and representative sample of hospital sections in the Capital Region of Denmark with a response rate of 84%, from which we included 148 hospital sections totalling 5205 employee responses. The detailed information on the composition of hospital sections and patient-level information enabled comprehensive adjustment for potential confounding at the level of both patients and employees. The analyses relied on 23,872 patient reports on validated measures of patient satisfaction, patient involvement, and medical errors regarding outcome measures. Relying on independently collected answers on the sides of the exposure (workplace social capital) and outcome variables (patient-reported quality of care) safeguards against common method bias [43]. For example, research shows that health care professionals’ willingness to report medical errors depends on the level of psychological safety [44].

Nevertheless, relying on cross-sectional data precludes longitudinal assessment of the relationship between workplace social capital and patient-reported quality of care. Furthermore, our study, similar to most other health care studies of social capital [20, 21, 29], primarily surveys one occupation-specific sample (nurses). This may limit the generalisability of the empirical evidence to other health care occupations working in hospitals, most notably the medical profession, which occupies a unique position due to its high level of professional autonomy [45]. However, all participants have reported on their collective work environment, including relations to all professions, including doctors. Further, since the survey is anonymous, it is unlikely that nurses would have reported a high level of social capital if their collaboration with doctors was strained. The study would have benefitted from including covariates such as staff-to-patient ratios and the type of medical speciality of each hospital section because they likely influence the psychosocial work environment and the quality of care health care professionals deliver to patients. Another limitation is the response rate (61% for inpatient, 45% for acute, and 56% for outpatient care) among the invited random sample of patients asked to report their patient experiences. These response rates could result in selection bias and influence the analysis if the responding and non-responding patients experienced different levels of care quality. Participation was generally lower for acute care, patients with a length-of-stay less than 24 h, and patients younger than 60 years. Relatedly, if the work context and environment differ between included and excluded types of hospital sections, the results may not apply to those types of sections excluded in this study (for example, where patient contact typically is a sub-part of the care pathway as is the case for diagnostic imaging, biochemistry, and nuclear medicine).

Our findings inform hospital management and policymakers, who increasingly understand patient quality as delivering patient-centred and coordinated care, in the following ways: moving hospital sections from having a section-level social capital belonging to the lowest 25% to the highest 25% (i.e., the IQR) would most likely result in an increase in patient-reported quality of care in most settings. Although there is a low variance in the distribution of section-level social capital, implementation of supportive collaborative practices would render it possible to start shifting the distribution and raise the level of social capital among a larger share of health care professionals. From the literature, relevant work-environment approaches include employing participatory workshops [46], joint collaborative committees [47], psychological safety [44], transformational leadership [48, 49], and supportive organisation designs [50]. That social capital in hospitals is associated with better quality management systems [32] and clinical risk management [33] amplifies why hospital management should pursue developing this inter-personal resource. Because workplace social capital is an organisational resource for employee well-being and the perceived quality of care for patients, our findings highlight the merits of investing health care managers’ and clinicians’ time in efforts to promote social capital among employees. This opens up for using work-place social capital to achieve a high level of care quality in hospitals that restructure and rationalise care delivery to meet calls for more and better health care.

## Conclusion

This study extends the existing literature on workplace social capital by analysing the effect of social capital across 148 Danish hospital sections spanning acute, inpatient, and outpatient care on patient-reported quality of care. Although we found small absolute differences in the association between social capital and overall patient satisfaction, patient involvement and patient-reported medical errors, even a small upward shift in the distribution of social capital in the hospital sector would at the population level have a large positive impact on patients’ care experience.

## Supplementary Information

**Additional file 1: Appendix 1.** Individual level patient characteristics among patients receiving care at NSPE hospital sections according to merge status with WHALE **Appendix 2.** Items covering section social capital and patient-reported quality of care. **Appendix 3a.** The correlation between section *trust* and patient-reported quality of care. **Appendix 3b.** The correlation between section *justice* and patient-reported quality of care. **Appendix 3c.** The correlation between section *collaboration* and patient-reported quality of care**. Appendix 4.** Dimensions of section social capital and mean differences in patient-reported quality of care in 148 hospital sections. **Appendix 5.** Dimensions of section social capital and risk ratios (95% CI) of being in the lowest tertile of patient-reported quality of care in 148 hospital sections.

## Data Availability

The data that support the findings of this study are available from the Capital Region of Denmark, but restrictions apply to the availability of these data, which were used under license for the current study, and so are not publicly available. Data are, however, available from the authors upon reasonable request and with permission of the Capital Region of Denmark.
