# Supplementary material for: The impact of work-place social capital in hospitals on patient-reported quality of care: a cohort study of 5205 employees and 23,872 patients in Denmark
Source: BMC Health Serv Res. 2021 May 31;21:534. doi: 10.1186/s12913-021-06498-x (PMC8167966; doi:10.1186/s12913-021-06498-x)
Supplement: Supplementary file 1 — Additional file 1: Appendix 1. Individual level patient characteristics among patients receiving care at NSPE hospital sections according to merge status with WHALE Appendix 2. Items covering section social capital and patient-reported quality of care. Appendix 3a. The correlation between section trust and patient-reported quality of care. Appendix 3b. The correlation between section justice and patient-reported quality of care. Appendix 3c. The correlation between section collaboration and patient-reported quality of care. Appendix 4. Dimensions of section social capital and mean differences in patient-reported quality of care in 148 hospital sections. Appendix 5. Dimensions of section social capital and risk ratios (95% CI) of being in the lowest tertile of patient-reported quality of care in 148 hospital sections. [file 12913_2021_6498_MOESM1_ESM.docx]

The impact of work-place social capital in hospitals on patient-reported quality of care: A cohort study of 5,205 employees and 23,872 patients in Denmark

Alice Clark

Department of Public Health,

University of Copenhagen

Thim Prætorius*

Steno Diabetes Center Aarhus,

Aarhus University Hospital

thipra@rm.dk

Eszter Török

Department of Public Health,

University of Copenhagen

Ulla Hvidtfeldt

Danish Cancer Society Research Center

Peter Hasle

Department of Technology and Innovation,

University of Southern Denmark

Naja Hulvej Rod

Department of Public Health,

University of Copenhagen

*corresponding author

Appendix 1. Individual level patient characteristics among patients receiving care at NSPE hospital sections according to merge status with WHALE

|  | **All** | **Merged**  **Hospital sections** | **Un-merged**  **Hospital sections** |
| --- | --- | --- | --- |
| **Number of patients** | 37,084 | 23,872 | 13,212 |
| **Patient satisfaction**, mean (SD) | 4.23 (0.85) | 4.21 (0.86) | 4.27 (0.83) |
| **Patient involvement**, mean (SD) | 3.61 (1.10) | 3.58 (1.10) | 3.68 (1.08) |
| **Medical errors**, mean proportion (SD) | 0.07 (0.26) | 0.08 (0.26) | 0.06 (0.25) |
| **Type of care** |  |  |  |
| Inpatient (%) | 6,294 (17) | 4,388 (18) | 1,906 (14) |
| Acute (%) | 7,708 (21) | 5,878 (25) | 1,830 (14) |
| Outpatient (%) | 23,082 (62) | 13,606 (57) | 9,476 (72) |
| **Gender** |  |  |  |
| Women (%) | 20,294 (55) | 12,686 (53) | 7,608 (58) |
| **Age** |  |  |  |
| ≥60 (%) | 21,070 (57) | 14,244 (60) | 6,826 (52) |
| **Lenght-of-stay^a^** | |  |  |
| ≥24 hours (%) | 8,041 (57) | 5,723 (56) | 2,318 (62) |

^a^ Not relevant for outpatient care.

**Appendix 2. Items covering section social capital and patient-reported quality of care.**

| **Social capital** |
| --- |
| *Trust* |
| To what degree does the management trust the employees to do their work well? (7) |
| To what degree do you trust the information that comes from the management? (7) |
| *Justice* |
| To what degree is the work distributed fairly? (7) |
| To what degree are conflicts resolved in a fair way? (7) |
| To what degree is your staff group respected by the other staff groups at the work-place? (5) |
| *Collaboration* |
| To what degree do you get help and support from your colleagues when needed? (5) |
| To what degree do you and your colleagues take responsibility for a nice atmosphere and tone of communication? (5) |
| To what degree are you and your colleagues good at coming up with suggestions for improving work procedures? (5) |
| **Patient reported quality of care** |
| *Patient satisfaction* |
| Are you satisfied with the care that you received? (5) |
| Are you satisfied with the treatment that you received for your disease/condition? (5) |
| Are you all in all satisfied with the stay, from when you were hospitalized until you were discharged? (5) |
| *Patient involvement* |
| Did the staff ask you for your own experiences with your disease/condition? (5) |
| Did the staff give you the opportunity to participate in decisions about your examination/treatment? (5) |
| Did the staff (after your consent) give your relatives the opportunity to participate in decisions about your examination/treatment? (5) |
| Did you have conversations with the staff about how to best manage your disease/condition? (5) |
| Did the staff consider your needs when planning your discharge? (5)* |
| *Medical errors* |
| Did you encounter any errors during your hospitalization/outpatient visit? (yes/no) |

Response categories on a 5-point (5) or a 7-point (7) Likert scale ranging from: ‘to a very large extent’ to ‘not at all. * Not applicable for outpatient care.

Section Trust

Section Trust

Section Trust

Inpatient

Outpatient

Acute

Section Trust

Section Trust

Section Trust

Inpatient

Acute

Outpatient

Section Trust

Section Trust

Section Trust

Inpatient

Outpatient

Acute

**Appendix 3a. The correlation between section *trust* and patient-reported quality of care**

Section Justice

Section Justice

Section Justice

Acute

Outpatient

Inpatient

Section Justice

Section Justice

Section Justice

Acute

Outpatient

Inpatient

Section Justice

Section Justice

Section Justice

Inpatient

Outpatient

Acute

**Appendix 3b. The correlation between section *justice* and patient-reported quality of care**

Section Collaboration

Section Collaboration

Section Collaboration

Section Collaboration

Section Collaboration

Section Collaboration

Acute

Outpatient

Inpatient

Inpatient

Outpatient

Acute

Section Collaboration

Section Collaboration

Section Collaboration

Inpatient

Outpatient

Acute

**Appendix 3c. The correlation between section *collaboration* and patient-reported quality of care**

###### Appendix 4. Dimensions of section social capital and mean differences in patient-reported quality of care in 148 hospital sections

|  | **Inpatient care** | **Acute care** | **Outpatient care** |
| --- | --- | --- | --- |
| Number of hospital sections | 44 | 60 | 78 |
| ***Section trust*** |  |  |  |
| Mean (IQR) hospital section trust | 72 (9) | 72 (7) | 75 (9) |
| **Mean patient satisfaction** |  |  |  |
| Mean level of patient satisfaction (SD) | 4.25 (0.19) | 4.04 (0.25) | 4.26 (0.16) |
| Multiple adjusted^a^ mean differences (95% CI) according to IQR of hospital section trust | 0.04 (-0.03, 0.12) | 0.05 (-0.01, 0.11) | -0.03 (-0.08, 0.02) |
| **Mean patient involvement** |  |  |  |
| Mean level of patient involvement (SD) | 3.62 (0.26) | 3.28 (0.35) | 3.75 (0.26) |
| Multiple adjusted^a^ mean differences (95% CI) according to IQR of hospital section trust | 0.12 (0.03, 0.21) | 0.05 (-0.02, 0.13) | 0.01 (-0.07, 0.08) |
| **Mean occurrence of medical errors** |  |  |  |
| Mean occurrence of medical errors (SD) | 0.09 (0.05) | 0.13 (0.06) | 0.05 (0.03) |
| Multiple adjusted^a^ mean differences (95% CI) according to IQR of hospital section trust | -0.01 (-0.03, 0.01) | -0.01 (-0.02, 0.02) | 0.00 (-0.01, 0.01) |
| ***Section justice*** |  |  |  |
| Mean (IQR) hospital section justice | 65 (6) | 65 (7) | 68 (8) |
| **Mean patient satisfaction** |  |  |  |
| Mean level of patient satisfaction (SD) | 4.25 (0.19) | 4.04 (0.25) | 4.26 (0.16) |
| Multiple adjusted^a^ mean differences (95% CI) according to IQR of hospital section justice | 0.06 (-0.02, 0.15) | 0.07 (-0.00, 0.14) | 0.02 (-0.05, 0.10) |
| **Mean patient involvement** |  |  |  |
| Mean level of patient involvement (SD) | 3.62 (0.26) | 3.28 (0.35) | 3.75 (0.26) |
| Multiple adjusted^a^ mean differences (95% CI) according to IQR of hospital section justice | 0.11 (-0.01, 0.22) | 0.08 (-0.01, 0.18) | 0.02 (-0.05, 0.10) |
| **Mean occurrence of medical errors** |  |  |  |
| Mean occurrence of medical errors (SD) | 0.09 (0.05) | 0.13 (0.06) | 0.05 (0.03) |
| Multiple adjusted^a^ mean differences (95% CI) according to IQR of hospital section justice | -0.01 (-0.03, 0.02) | -0.01 (-0.03, 0.02) | 0.00 (-0.01, 0.01) |
| ***Section collaboration*** |  |  |  |
| Mean (IQR) hospital section collaboration | 72 (6) | 71 (6) | 74 (7) |
| **Mean patient satisfaction** |  |  |  |
| Mean level of patient satisfaction (SD) | 4.25 (0.19) | 4.04 (0.25) | 4.26 (0.16) |
| Multiple adjusted^a^ mean differences (95% CI) according to IQR of hospital section collaboration | 0.09 (-0.00, 0.19) | 0.10 (0.01, 0.19) | 0.01 (-0.05, 0.06) |
| **Mean patient involvement** |  |  |  |
| Mean level of patient involvement (SD) | 3.62 (0.26) | 3.28 (0.35) | 3.75 (0.26) |
| Multiple adjusted^a^ mean differences (95% CI) according to IQR of hospital section collaboration | 0.06 (-0.08, 0.20) | 0.08 (-0.04, 0.19) | 0.01 (-0.08, 0.09) |
| **Mean occurrence of medical errors** |  |  |  |
| Mean occurrence of medical errors (SD) | 0.09 (0.05) | 0.13 (0.06) | 0.05 (0.03) |
| Multiple adjusted^a^ mean differences (95% CI) according to IQR of hospital section collaboration | 0.00 (-0.03, 0.03) | -0.02 (-0.05, 0.01) | 0.01 (-0.00, 0.02) |

^a^Adjusted for section characteristics: Hospital, number of employees, complexity, mean age of employees, proportion of females, proportion of part-time employees, proportion with prior LTSA, patient characteristics: proportion females, proportion 60 years or older, proportion with length-of-stay exceeding 24 hours.

Appendix 5. Dimensions of section social capital and risk ratios (95% CI) of being in the lowest tertile of patient-reported quality of care in 148 hospital sections

|  | **Inpatient care** | **Acute care** | **Outpatient care** |
| --- | --- | --- | --- |
| Number of hospital sections | 44 | 60 | 78 |
| ***Section trust*** |  |  |  |
| Mean (IQR) hospital section trust | 72 (9) | 72 (7) | 75 (9) |
| **Patient satisfaction** |  |  |  |
| Lowest 33-percentile cut-point of patient satisfaction | 4.19 | 3.96 | 4.20 |
| Multiple adjusted^a^ RR (95% CI) according to IQR of hospital section trust | 0.62 (0.42, 0.90) | 0.44 (0.29, 0.66) | 1.08 (0.70, 1.64) |
| **Patient involvement** |  |  |  |
| Lowest 33-percentile cut-point of patient involvement | 3.49 | 3.10 | 3.63 |
| Multiple adjusted^a^ RR (95% CI) according to IQR of hospital section trust | 0.67 (0.38, 1.18) | 0.67 (0.37, 1.23) | 0.73 (0.51, 1.04) |
| **Medical errors** |  |  |  |
| Highest 33-percentile cut-point of the occurrence of medical errors | 0.11 | 0.14 | 0.06 |
| Multiple adjusted^a^ RR (95% CI), according to IQR of hospital section trust | 0.84 (0.59, 1.18) | 0.74 (0.52, 1.07) | 1.21 (0.78, 1.86) |
| ***Section justice*** |  |  |  |
| Mean (IQR) hospital section justice | 65 (6) | 65 (7) | 68 (8) |
| **Patient satisfaction** |  |  |  |
| Lowest 33-percentile cut-point of patient satisfaction | 4.19 | 3.96 | 4.20 |
| Multiple adjusted^a^ RR (95% CI) according to IQR of hospital section justice | 0.60 (0.28, 1.28) | 0.44 (0.24, 0.82) | 0.84 (0.53, 1.33) |
| **Patient involvement** |  |  |  |
| Lowest 33-percentile cut-point of patient involvement | 3.49 | 3.10 | 3.63 |
| Multiple adjusted^a^ RR (95% CI) according to IQR of hospital section justice | 0.53 (0.30, 0.92) | 0.59 (0.36, 0.97) | 0.89 (0.53, 1.50) |
| **Medical errors** |  |  |  |
| Highest 33-percentile cut-point of the occurrence of medical errors | 0.11 | 0.14 | 0.06 |
| Multiple adjusted^a^ RR (95% CI), according to IQR of hospital section justice | 0.94 (0.58, 1.50) | 0.72 (0.48, 1.09) | 1.03 (0.65, 1.63) |
| ***Section collaboration*** |  |  |  |
| Mean (IQR) hospital section collaboration | 72 (6) | 71 (6) | 74 (7) |
| **Patient satisfaction** |  |  |  |
| Lowest 33-percentile cut-point of patient satisfaction | 4.19 | 3.96 | 4.20 |
| Multiple adjusted^a^ RR (95% CI) according to IQR of hospital section collaboration | 1.72 (0.84, 3.51) | 0.72 (0.41, 1.26) | 0.73 (0.45, 1.19) |
| **Patient involvement** |  |  |  |
| Lowest 33-percentile cut-point of patient involvement | 3.49 | 3.10 | 3.63 |
| Multiple adjusted^a^ RR (95% CI) according to IQR of hospital section collaboration | 0.49 (0.09, 2.62) | 0.45 (0.26, 0.75) | 1.37 (0.81, 2.32) |
| **Medical errors** |  |  |  |
| Highest 33-percentile cut-point of the occurrence of medical errors | 0.11 | 0.14 | 0.06 |
| Multiple adjusted^a^ RR (95% CI), according to IQR of hospital section collaboration | 1.10 (0.41, 2.95) | 0.62 (0.32, 1.21) | 1.29 (0.84, 1.98) |

^a^Adjusted for section characteristics: Hospital, number of employees, complexity, mean age of employees, proportion of females, proportion of part-time employees, proportion with prior LTSA, patient characteristics: proportion females, proportion 60 years or older, proportion with length-of-stay exceeding 24 hours.
